# Clinical factors associated with septic shock in pediatric invasive meningococcal disease: a retrospective study from two tertiary hospitals in Chile, 2009–2025

**DOI:** 10.3389/fpubh.2026.1911106

**Published:** 2026-10-13

**Authors:** Cindy Arteta-Acosta, Maritza Márquez, Rodolfo Villena, María Elena Santolaya

**Affiliations:** 1 CICA Hospital de niños Dr. Luis Calvo Mackenna, Department of Pediatrics, Faculty of Medicine, Universidad de Chile, Santiago, Chile; 2 Pontificia Universidad Católica de Chile, Faculty of Mathematics, Universidad Católica de Chile, Santiago, Chile; 3 Department of Pediatrics, CICA Hospital de niños Dr. Exequiel González Cortes, Faculty of Medicine, Universidad de Chile, Santiago, Chile

**Keywords:** meningitis, meningococcal infections, mortality, pediatrics, septic shock

## Abstract

**Background:**

Invasive meningococcal disease (IMD) remains a primary etiology of bacterial meningitis and a cause of long-term neurological sequelae in children. We aimed to describe characteristics of IMD and to identify variables associated with adverse outcomes, defined as septic shock.

**Methods:**

A retrospective cohort study included children with microbiologically confirmed IMD hospitalized at two tertiary pediatric hospitals in Santiago, Chile (2009–2025). Data were collected from medical records. Variables associated with the outcome were assessed using Firth’s penalized logistic regression, with candidate variables selected *a priori* according to clinical relevance, availability at admission, and absence of overlapping with the outcome process.

**Results:**

Seventy-two children were included; 65.3% were male and 54.2% were infants younger than 12 months. Over the 17-year study period, most cases occurred between 2009 and 2014 (*n* = 51), followed by a marked decline from 2015 to 2025 (*n* = 21) coinciding with the introduction of the meningococcal ACWY vaccine in the National Immunization Program. The most frequent manifestations at admission were toxic appearance (79.2%), fever (69.4%), gastrointestinal symptoms (69.4%), and irritability or drowsiness (62.5%). The main diagnoses were meningitis with meningococcemia (37.5%), isolated meningitis (25.0%), and meningococcemia (25.0%). Serogroup W (50.0%) and serogroup B (36.1%) predominated. Septic shock occurred in 28 children (38.9%), with a case-fatality rate of 6.9% (5/72); all deaths occurred in children with meningococcemia and none in isolated meningitis. In the primary multivariable model, seizure during hospitalization (aOR 10.63; 2.33–63.90), prolonged capillary refill (aOR 5.75; 1.49–26.21), and petechial or purpuric rash (aOR 4.47; 1.24–18.87), were associated with adverse outcome.

**Conclusion:**

Pediatric IMD remains an important cause of severe disease and mortality. The factors associated with severe outcome reflected three clinically relevant domains: neurological involvement, impaired peripheral perfusion, and vascular manifestations. Because these are simple clinical signs recognizable without laboratory testing, they may support early risk stratification and timely intervention, particularly in resource-limited settings.

## 1 Introduction

Invasive meningococcal disease (IMD) is an infectious disease caused by *Neisseria meningitidis* classified into 12 serogroups, of which six are the most invasive and cause most of the disease burden (A, B, C, W, X, and Y) (1). The epidemiology of IMD is dependent on the circulating serogroups, vaccination strategies, and population immunity. Globally, more than 400,000 cases of meningococcal meningitis occur annually, with an estimated case-fatality rate (CFR) of approximately 8% (2). Prior to 2020, a decline in incidence of IMD was reported due to the introduction of meningococcal conjugate vaccine against serogroups A, C, W and Y (MCV-ACWY) and the four-component meningococcal vaccine (4CMenB). Additionally, the COVID-19 pandemic was associated with a temporary reduction in transmission across multiple countries related to prevention measures adopted during this period (3–6). However, disruptions to routine immunization programs during this period facilitated a resurgence of IMD in 2021–2022, particularly serogroup B, among unvaccinated cohorts (5–7).

In Chile, strict surveillance mandates the immediate notification of suspected cases of IMD to the local Epidemiology Department of the Ministry of Health, and microbiological confirmation of *N. meningitidis* by the Public Health Institute of Chile (8). In 2014, the MCV-ACWY was introduced into the National Immunization Program (NIP) for infants of 12 months of age. Later, in 2023, the 4CMenB vaccine was introduced for infants at 2 and 4 months of age with a booster dose at 18 months (9). The current incidence rate has stabilized at pre-pandemic levels at 0.5 cases per 100,000 inhabitants. The serogroup distribution is characterized by a predominance of serogroup W (MenW, 39%) and serogroup B (MenB, 34%), with an increasing proportion of serogroups C (MenC, 9%) and Y (MenY, 8%) (10, 11).

IMD has a rapid and often unpredictable course, especially in previously healthy children and adolescents (2). It typically presents as meningitis, meningococcemia, or a combination of both; meningitis is the most frequent clinical presentation, and meningococcemia forms are associated with the highest CFR, ranging between 10 and 25%, reaching 50% in the absence of intervention, with sequelae in up to 60% of survivors (12–14). The poor prognosis in pediatric IMD has been associated with a combination of factors including hemodynamic and respiratory involvement, neurological compromise, metabolic alterations, and hematological abnormalities at hospital admission (15, 16), reflecting the pathophysiology of meningococcal shock, which is characterized by endothelial dysfunction, coagulopathy, tissue hypoperfusion, and a dysregulated systemic inflammatory response (12). The clinical heterogeneity of IMD, ranging from isolated meningitis to septic shock, poses significant challenges for recognition and risk stratification (15, 16).

In this context, the early recognition of IMD and identification of factors associated with outcomes are critical to improve survival and prognosis. Therefore, the aim of this study was to describe the clinical and laboratory characteristics, as well as factors associated with adverse outcome, defined as septic shock, in pediatric patients with IMD treated at two pediatric public hospitals in Santiago, Chile, between 2009 and 2025.

## 2 Materials and methods

### 2.1 Study design

A retrospective cohort study was carried out in patients under 15 years of age, hospitalized for IMD between 2009 and 2025 at two public pediatric hospitals in Santiago, Chile: Hospital Dr. Luis Calvo Mackenna, a referral center for the Metropolitan East Health Service and Hospital Dr. Exequiel González Cortés a referral center for the Metropolitan South Health Service. The study was approved by the Ethics Committee for Research of the Faculty of Medicine, Universidad de Chile.

### 2.2 Study subjects and definitions

Patients with confirmed IMD, with compatible clinical presentation plus *N. meningitidis* identified by culture and/or real-time polymerase chain reaction (RT-PCR) targeting the *ctrA gene* from normally sterile body fluid or tissue were included. Isolates and specimens from culture-negative suspected cases were referred to the national reference laboratory (Instituto de Salud Pública, ISP) for confirmation and serogroup determination. Serogroup was determined by slide agglutination and, since 2013, by RT-PCR, under the national surveillance framework (8, 17).

*Meningococcemia* was defined as a positive culture and/or PCR for *N. meningitidis* from blood with a compatible clinical presentation. *Meningitis* was defined as positive culture and/or PCR from cerebrospinal fluid (CSF) (18). *Occult bacteremia* was defined as a positive blood culture of a well-appearing febrile child without focus of infection. *Meningitis with meningococcemia* was defined as positive culture and/or PCR in blood and CSF (18). *Septic arthritis* was defined as identification of *N. meningitidis* in synovial fluid (18). *Septic shock* was defined, according to the “international Surviving Sepsis Campaign guidelines”, as severe infection leading to cardiovascular dysfunction, including hypotension, the need for vasoactive drugs (VAD), or impaired perfusion (19).

### 2.3 Variable measures

The primary outcome was the proportion of patients who developed septic shock during IMD hospitalization. This outcome was selected to capture severe clinical deterioration and to support a more stable multivariable analysis. As independent covariates we included sociodemographic characteristics (age in months, sex, and socioeconomic status); nutritional status (overweight, normal nutritional status, and underweight); comorbidity (recurrent wheezing or asthma, prematurity, immunodeficiency, congenital cardiopathy, neurological disease, genetic and other disorders); signs and symptoms (fever, toxic clinical appearance, hypotension, hypoxemia, Glasgow Coma Scale (GCS) at admission, vomiting, diarrhea, abdominal pain, headache, meningeal signs [neck stiffness, positive Kernig or Brudzinski signs, and bulging fontanelle in infants with open fontanelle], focal neurologic deficit, irritability, drowsiness, petechial or purpuric rash, respiratory symptoms, prolonged capillary refill, seizures at admission and seizures during hospitalization [included seizures occurring at any point from admission onward]); clinical diagnosis (meningitis, meningococcemia, meningitis with meningococcemia, occult bacteremia, and septic arthritis); hospitalization variables (time of onset of symptoms, hospitalization, mechanical ventilation, VAD, pediatric intensive care unit (PICU) admission); severe systemic manifestations (adrenal insufficiency, limb ischemia, amputation, multiorgan failure); and laboratory variables (C reactive protein, hemogram, lactate, serogroups, cultures, and PCR).

### 2.4 Statistical analysis

Categorical variables were summarized as absolute frequencies and percentages. Continuous variables were described using means and standard deviations (SD) or medians and interquartile ranges [IQR, 25th–75th percentiles], according to their distribution as assessed by the Shapiro–Wilk test. Comparisons between clinical groups were performed using the chi-square test or Fisher’s exact test for categorical variables, and Student’s t-test or the Mann–Whitney U test for continuous variables, as appropriate. Univariable comparisons were considered exploratory and were not adjusted for multiple testing. No *p*-value threshold was used as an automatic screening criterion for entry into multivariable models. Variables were not excluded solely because they lacked statistical significance in univariable analysis, nor were statistically significant variables included automatically when they showed temporal overlap with the outcome, substantial missingness, low frequency, or conceptual redundancy.

Candidate variables for the multivariable models were selected according to clinical relevance, evidence from previous studies, availability at hospital admission, data completeness, temporal relationship with the outcome, and conceptual overlap among variables. Variables arising during hospitalization or closely overlapping with the outcome process were not considered for the primary model and were examined only in secondary analyses. Redundancy and collinearity were assessed using phi correlations, contingency tables, and variance inflation factors (VIF) (Supplementary Figure S1). Data completeness was evaluated for each candidate variable, and variables with missing information were reported using the available denominator (Supplementary Figure S2). Based on these criteria, a parsimonious primary model was defined using complete clinical variables. Additional variables that could plausibly be associated with the outcome, including clinical presentation and microbiological characteristics, were evaluated in an extended secondary model.

Adjusted associations with the outcome were estimated using Firth’s penalized logistic regression (20). Conventional maximum-likelihood logistic regression was initially assessed; however, sparse cells and complete or quasi-complete separation produced unstable or infinite estimates for some variables. Firth regression was therefore used to reduce small-sample bias and obtain finite estimates under separation. Adjusted odds ratios (aOR) and 95% confidence intervals (95% CIs) were obtained from the profile penalized likelihood.

Sensitivity analyses included refitting alternative model specifications in the full cohort, applying multiple imputation for variables with missing data, using Akaike information criterion (AIC)-based forward selection, alternative bias-reduction and assessing model stability through bootstrap and leave-one-out analyses.

Analyses were conducted in R version 4.6.0 (21) using the *logistf* package (version 1.26.1) for Firth regression. Two-sided *p* values and 95% CIs are reported. Analysis code was archived to support reproducibility.

## 3 Results

### 3.1 Study population

During the study period, a total of 72 children were hospitalized with IMD. Over the 17-year study period, 70.8% of cases occurred between 2009 and 2014, followed by a marked decline from 2015 to 2025 (29.2%) (Figure 1). Median age was 9 months [IQR 4.0–34.5], 65.3% were male, and 54.2% were infants younger than 12 months of age. Approximately 20.8% were overweight/obesity and 13.9% were underweight/risk. Thirty-eight percent had some comorbidity and 38.9% belonged to low socioeconomic status (Table 1).

**Figure 1:**
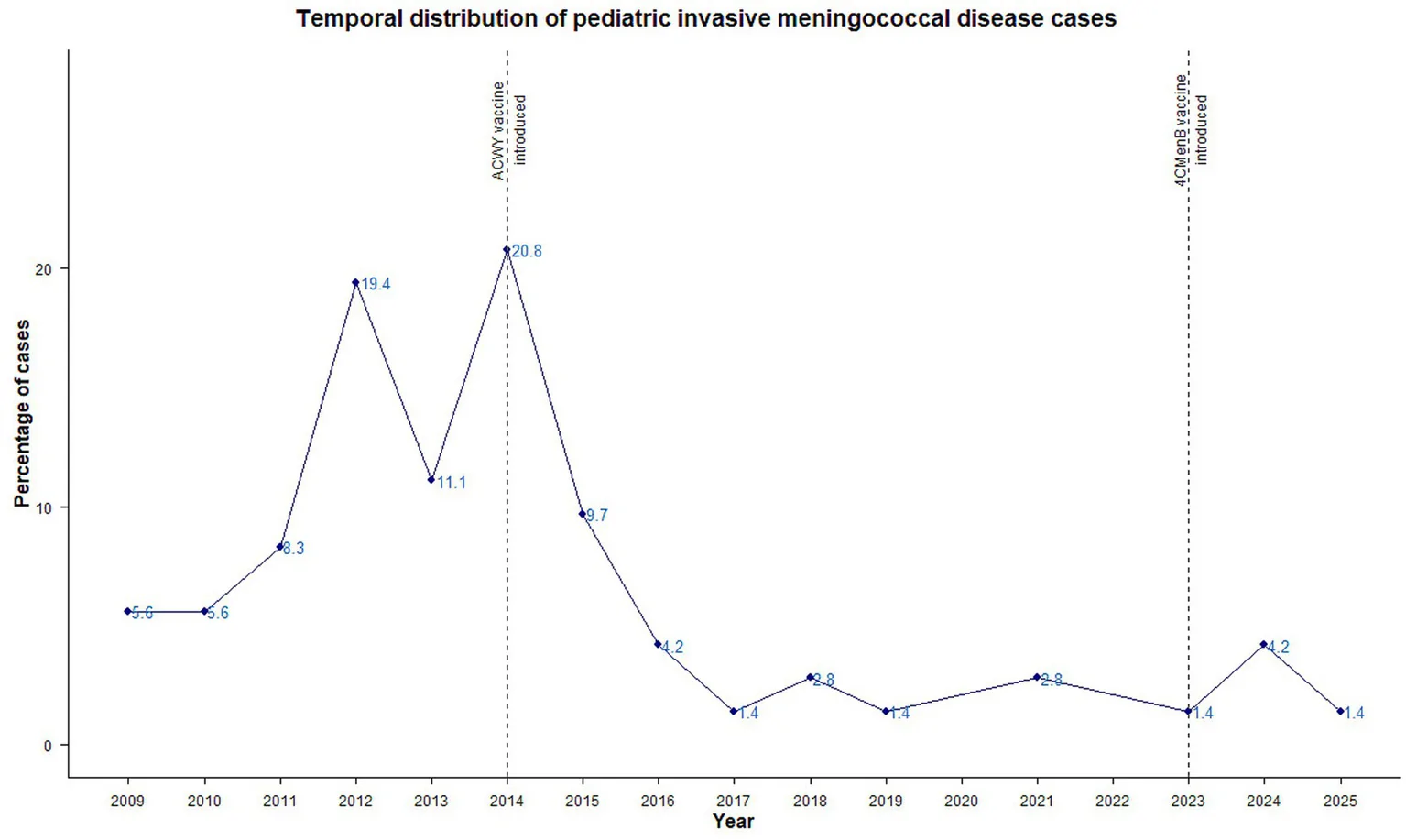
Temporal distribution of pediatric invasive meningococcal disease between 2009–2025, Santiago Chile.

**Table 1:** Sociodemographic characteristics of children with invasive meningococcal disease between 2009–2025, Santiago Chile.

Variables		*N* = 72 (%)
Median age (months), [pc 25–75]		9.00 [4.00–34.50]
Age (months), mean ± SD		29.40 ± 40.16
Age younger than 12 months of age, *n* (%)		39 (54.2)
Male sex, *n* (%)		47 (65.3)
Nutritional status, *n* (%)	Overweight/obesity	15 (20.8)
	Normal nutritional status	45 (62.5)
	Undernutrition/risk	10 (13.9)
	No information	2 (2.8)
Comorbidity, *n* (%)	Any comorbidity	28 (38.9)
	Respiratory	13 (18.1)
	Preterm	3 (4.2)
	Preterm and neurology	2 (2.8)
	Immunodeficiency	2 (2.8)
	Congenital cardiopathy	1 (1.4)
	Neurodegenerative disease	1 (1.4)
	Genetics	1 (1.4)
	Sickle cell anemia	1 (1.4)
	Others	4 (5.6)
Socioeconomic status, *n* (%)	High	1 (1.4)
	Middle	43 (59.7)
	Low	28 (38.9)

IMD was suspected at the first emergency department visit in 15.3% of patients. The median time from symptom onset to hospital admission and initiation of antibiotic therapy was 2 days. The median duration of antibiotic treatment was 7 days [IQR 7.0–10.0]. Hospital length of stay showed substantial variability, with a median of 8 days [IQR 6.0–13.0] and a mean of 11.08 ± 11.0 SD days. Seventy-six percent of patients required admission to a PICU. Among these patients, the median length of stay was 3 days [IQR 2.0–4.0]. Mechanical ventilation was required in 26 patients (36.1%), although the median duration was less than 24 h [IQR 1.2–4.0], reflecting short-term use in most cases. VAD were administered to 20 patients (27.8%), with a median duration of 2.5 days [IQR 1.0–3.0].

### 3.2 Clinical characterization and outcome

At hospital admission, most children presented with a toxic clinical appearance (79.2%), fever (69.4%), and gastrointestinal symptoms (69.4%). Altered mental status, manifested as irritability or drowsiness, was observed in 62.5%. The median GCS score at admission was 14 [IQR 13–15]. Meningeal signs were present in 51.4% and meningococcal rash in 41.7%. The most frequent clinical presentation was meningitis with meningococcemia (37.5%), followed by meningitis (25.0%) and meningococcemia (25.0%). Septic arthritis and occult bacteremia were less common (Table 2).

**Table 2:** Clinical factors associated with septic shock in children with invasive meningococcal disease between 2009–2025, Santiago Chile.

Variable		Total, *N* = 72	Septic shock or death, *N* = 28 (%)	No shock or death, *N* = 44 (%)	*p*-value
Age younger 12 months of age, *n* (%)		39 (54.2)	14 (50.0)	25 (56.8)	0.746*
Male sex, *n* (%)		47 (65.3)	18 (64.3)	29 (65.9)	1.000*
Signs and symptoms					
Glasgow coma scale, median [IQR 1–3]		14 [13–15]	13 [11–14]	15 [14–15]	<0.001#
Toxic clinical appearance, *n* (%)		57 (79.2)	27 (96.4)	30 (68.2)	0.010*
Fever at hospital admission, *n* (%)		50 (69.4)	21 (75.0)	29 (65.9)	0.579*
Any gastrointestinal symptoms, *n* (%)		50 (69.4)	19 (67.8)	31 (70.4)	1.000*
Irritability or drowsiness, *n* (%)		45 (62.5)	25 (89.3)	20 (45.4)	<0.001*
Vomiting, *n* (%)		43 (59.7)	17 (60.7)	26 (59.0)	1.000*
Respiratory symptoms, *n* (%)		39 (54.2)	17 (60.7)	22 (50.0)	0.518*
Meningeal signs, *n* (%)		37 (51.4)	19 (67.8)	18 (40.9)	0.047*
Hypotension, *n* (%)		31 (43.1)	18 (64.3)	13 (29.5)	0.007*
Petechial or purpuric rash, *n* (%)		30 (41.7)	17 (60.7)	13 (29.5)	0.017*
Comorbidity, *n* (%)		28 (38.9)	11 (39.3)	17 (38.6)	1.000*
Focal neurological deficit, *n* (%)		26 (36.1)	15 (53.6)	11 (25.0)	0.027*
Prolonged capillary refill, *n* (%)		26 (36.1)	18 (64.3)	8 (18.2)	<0.001*
Headache, *n* (%)		19 (26.4)	9 (32.1)	10 (22.7)	0.542*
Seizures during hospitalization^&^, *n* (%)		19 (26.4)	12 (42.9)	7 (16.0)	0.015**
Diarrhea, *n* (%)		17 (23.6)	5 (17.8)	12 (27.3)	0.527*
Seizures at admission, *n* (%)		14 (19.4)	11 (39.3)	3 (6.8)	0.002*
Abdominal pain, *n* (%)		9 (12.5)	3 (10.7)	6 (13.6)	1.000**
Multiorgan failure		8 (11.1)	8 (28.6)	0 (0)	<0.001**
Hypoxemia, *n* (%)		8 (11.1)	6 (21.4)	2 (4.5)	0.049**
Limb ischemia, *n* (%)		8 (11.1)	7 (25.0)	1 (2.3)	<0.001**
Adrenal insufficiency, *n* (%)		4 (5.6)	4 (14.3)	0 (0)	0.019**
Clinical diagnosis					
Meningitis with Meningococcemia, *n* (%)		27 (37.5)	18 (64.3)	9 (20.4)	0.001**
Meningococcemia, *n* (%)		18 (25.0)	6 (21.4)	12 (27.2)	
Meningitis, *n* (%)		18 (25.0)	4 (14.3)	14 (31.8)	
Septic arthritis, *n* (%)		7 (9.7)	0 (0)	7 (15.9)	
Occult bacteremia, *n* (%)		2 (2.8)	0 (0)	2 (4.5)	
Laboratory variables					
Leukocytosis > 20,000 cells, n/N (%)		26/71 (36.6)	8/27 (29.6)	18/44 (40.9)	0.481*
Lactate > 20 mg/dL, n/N (%)		24/48 (50.0)	16/23 (69.6)	8/25 (32.0)	0.020*
Thrombocytopenia, n/N (%)		15/70 (21.4)	8/27 (29.6)	7/43 (16.3)	0.305*
Leukopenia, n/N (%)		6/71 (8.5)	4/27 (14.8)	2/44 (4.5)	0.192**
Serogroup, n 64 (%)	B	26 (36.1)	16 (57.1)	10 (22.7)	0.006*
	C	1 (1.4)	0 (0)	1 (2.3)	-
	W	36 (50)	9 (32.1)	27 (61.4)	0.029*
	Y	1 (1.4)	0 (0)	1 (2.3)	-
	Not data	8 (11.1)	3 (10.7)	5 (11.4)	-
PICU admission, *n* (%)		55 (76.4)	28 (100)	27 (61.3)	<0.001*
Mechanical ventilation, *n* (%)		26 (36.1)	26 (92.9)	0 (0)	<0.001**
Vasoactive drugs, *n* (%)		20 (27.8)	20 (71.4)	0	<0.001**

PICU, Pediatric Intensive Care Unit; IQR, interquartile range. Categorical variables: *chi-square test with Yates’ correction, **Fisher’s exact test (used when expected frequency <5); #Mann–Whitney U test for continuous. ^&^Seizures during hospitalization, included seizures occurring at any point from admission onward.

Twenty-eight children (38.9%) developed septic shock, five of them died (CFR 6.9%), two within the first 24 h of admission. When comparing patients who developed septic shock (*n* = 28) with those who did not (*n* = 44), markers of systemic severity were significantly more frequent among those with adverse outcomes. These included toxic clinical appearance (96.4% vs. 68.2%, *p* = 0.010), irritability or drowsiness (89.3% vs. 45.4%, *p* < 0.001), hypotension (64.3% vs. 29.5%, *p* = 0.007), and prolonged capillary refill (64.3% vs. 18.2%, *p* < 0.001). Consistent with the higher frequency of irritability or drowsiness, the GCS score at admission was lower in patients with adverse outcomes (median 13 [IQR 11–14] vs. 15 [IQR 14–15], respectively; *p* < 0.001). Neurological and perfusion-related complications were also more common, including focal neurological deficits (53.6% vs. 25.0%, *p* = 0.027), seizures at admission (39.3% vs. 6.8%, *p* = 0.002) and seizures during hospitalization (42.9% vs. 16.0, *p* = 0.015), meningeal signs (67.8% vs. 40.9%, *p* = 0.047), petechial or purpuric rash (60.7% vs. 29.5%, *p* = 0.017), limb ischemia (25.0% vs. 2.3%, *p* < 0.001), acute adrenal insufficiency (14.3% vs. 0%, *p* = 0.019), and multiorgan failure (28.6% vs. 0%, *p* < 0.001) (Table 2).

Regarding clinical presentation, meningitis with meningococcemia was more frequent among patients with septic shock (64.3% vs. 20.4%), whereas isolated meningitis predominated in those without adverse outcomes (31.8% vs. 14.3%). All patients with septic shock required PICU admission, compared with 61.3% of those without adverse outcomes (*p* < 0.001) (Table 2).

Symptom distribution varied markedly by clinical presentation (Figure 2). Patients with meningitis with meningococcemia had a severe systemic profile, characterized by toxic appearance (96.3%), irritability or drowsiness (81.5%), hypotension (59.3%), prolonged capillary refill (55.6%), petechial or purpuric rash (55.6%), septic shock (51.9%), and the highest CFR (14.8%). In contrast, patients with isolated meningitis showed a more localized clinical pattern, characterized predominantly by classical meningeal manifestations rather than systemic compromise. At the moment of admission, 94.4% of patients had fever, and meningeal signs were frequent in 77.8%. Headache and altered mental status were also common, whereas markers of circulatory failure and shock were less frequent compared with meningococcal presentations. Importantly, no deaths occurred among patients with isolated meningitis. Patients with meningococcemia without meningitis displayed an intermediate pattern, with prominent systemic involvement including respiratory symptoms (66.7%), hypotension (50.0%), petechial or purpuric rash (44.4%), and septic shock (27.8%), and a lower CFR (5.6%) compared with the combined presentation. Gastrointestinal symptoms were common across all three main clinical groups and occurred in similar proportions, indicating limited discriminatory value between presentations.

**Figure 2:**
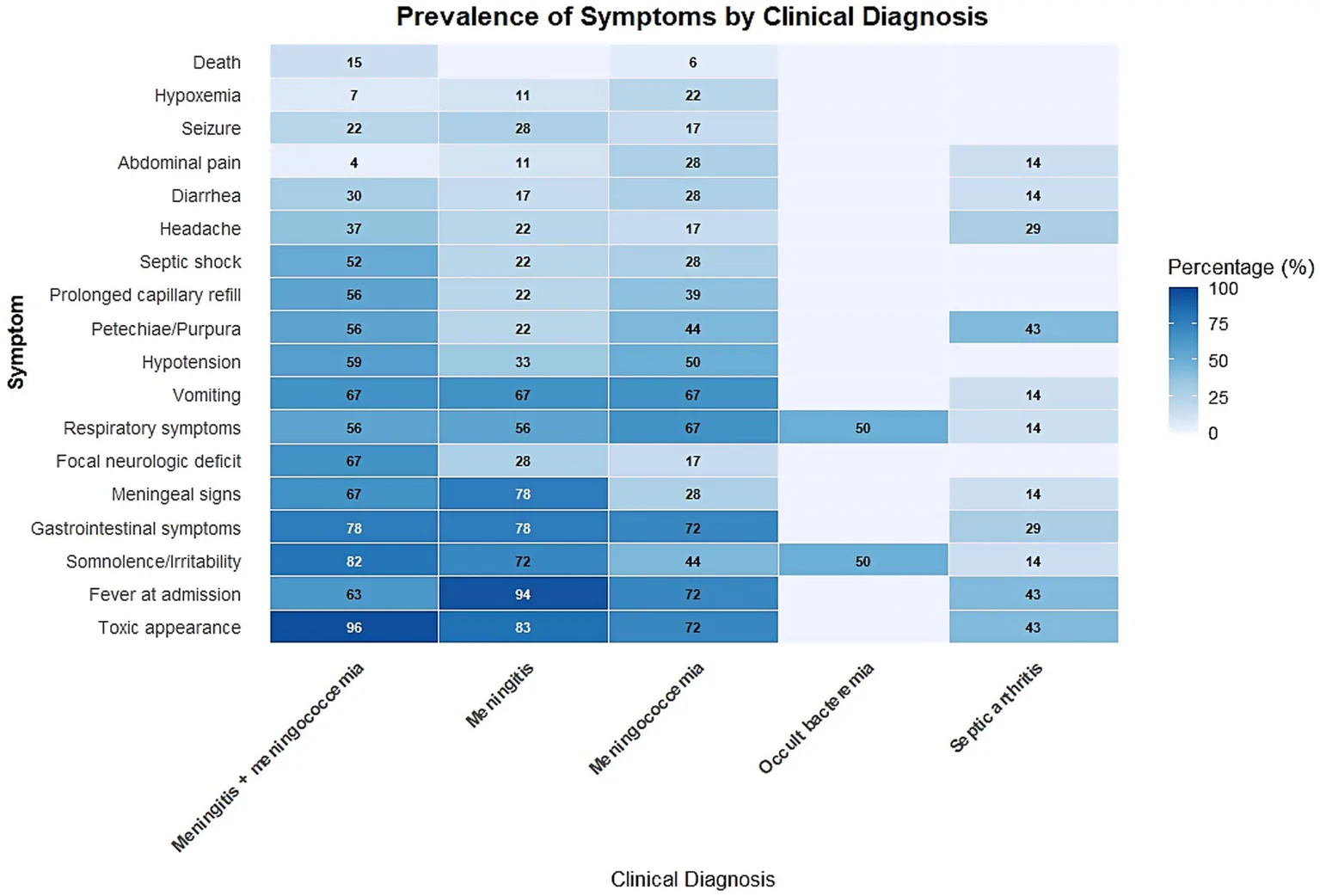
Distribution of clinical manifestation by clinical diagnosis in pediatric invasive meningococcal disease between 2009–2025, Santiago Chile.

### 3.3 Microbiological and laboratory findings

Of seventy-two cases included, 58 (80.6%) were confirmed by culture alone, 6 (8.3%) by PCR alone, and 8 (11.1%) by both methods. Samples were obtained from blood in 29 cases (40.3%), CSF in 22 (30.6%), both blood and CSF in 15 (20.8%), and synovial fluid in 6 (8.3%). MenW predominated (36/72, 50.0), followed by MenB (26/72, 36.1%), whereas MenC and MenY were identified in one patient each (1.4%); serogroup information was not available in 8 patients (11.1%).

Among laboratory parameters, lactate was measured in 48/72 children (66.7%) at the clinician’s discretion rather than systematically. Elevated lactate (>20 mg/dL) was more frequent in children with adverse outcome (16/23 [69.6%] vs. 8/25 [32.0%], *p* = 0.020), whereas leukocytosis, leukopenia, and thrombocytopenia did not differ significantly between groups (Table 2).

### 3.4 Factors associated with septic shock

The primary Firth model was restricted to four clinical variables and included all 72 patients with 28 adverse outcomes. Seizures during hospitalization (aOR 10.63; 95% CI 2.33–63.90; *p* = 0.002), prolonged capillary refill (aOR 5.75; 95% CI 1.49–26.21; *p* = 0.011) and petechial or purpuric rash (aOR 4.47; 95% CI 1.24–18.87; *p* = 0.021), were associated with higher odds of septic shock. Irritability or drowsiness had an aOR of 2.57 (95% CI 0.58–12.67; *p* = 0.212d) after adjustment (Table 3).

**Table 3:** Factors associated with adverse outcome pediatric invasive meningococcal disease between 2009–2025, Santiago Chile.

Variable	aOR*	95% CI	*p*-value
Seizure during hospitalization	10.63	2.33–63.90	0.002
Prolonged capillary refill	5.75	1.49–26.21	0.011
Petechial or purpuric rash	4.47	1.24–18.87	0.021
Irritability/drowsiness	2.57	0.58–12.67	0.212

*Adjusted odds ratios from the primary Firth-penalized logistic regression restricted to variables recorded during hospitalization. The model included 72 patients and 28 adverse outcomes; 95% CIs were obtained from the profile penalized likelihood.

In the extended model, the directions and magnitudes of the associations for seizure during hospitalization (aOR 15.34; 95% CI 2.86–122.42), and prolonged capillary refill (aOR 5.71; 95% CI 1.31–31.16) were similar. The estimates for irritability or drowsiness (aOR 1.95; 95% CI 0.37–10.81), petechial or purpuric rash (aOR 2.88; 95% CI 0.76–12.16), isolated meningitis (aOR 0.21; 95% CI 0.03–1.05) and serogroup B (aOR 2.77; 95% CI 0.72–11.55) were imprecise and compatible with no adjusted association, Supplementary Table S1.

Sensitivity analyses supported the direction of the main associations while showing substantial uncertainty in their magnitude. In the alternative model, prolonged capillary refill (aOR 6.64 95% CI 1.62–33.07), petechial or purpuric rash (aOR 6.71; 95% CI 1.70–33.64), and seizure at admission (aOR 23.98; 95% CI 4.07–203.77) remained associated with the outcome (Supplementary Table S1). Multiple imputation for lactate suggested a positive but imprecise association for lactate >20 mg/dL, without materially changing the direction of the principal clinical coefficients. Alternative leave-one-patient-out analyses preserved those directions (Supplementary Table S1), whereas bootstrap resampling showed that stepwise AIC selection was not fully stable.

## 4 Discussion

The present retrospective cohort study included children with confirmed IMD over a period of 17 years in Chile. More than half of the patients were infants, and 28/72 experienced septic shock. Nevertheless, age younger than 12 months was not associated with the outcome in the exploratory analysis, indicating that the high representation of infants in the cohort should not be interpreted as an independent severity effect. This age distribution is consistent with international epidemiological data showing that the incidence of IMD is generally highest during the first year of life (22), owing to immunological immaturity and the absence of vaccine protection at this age during most of the study period. This early susceptibility, together with the rapidly progressive nature of IMD, contributes to the substantial morbidity and mortality reported despite advances in antimicrobial therapy, intensive care, and vaccination (3, 12, 23).

The overall CFR observed in our patients was 6.9%, comparable to that reported in high-income countries, where pediatric IMD fatality rates range between 5 and 10%, but lower than those described in low- and middle-income settings, including other Latin American countries (24–26).

Historical studies have identified factors associated with mortality in IMD. A Brazilian study identified thrombocytopenia <100,000/mm^3^ (aOR 13.8; 95% CI 3.1–60.9), metabolic acidosis (aOR 6.0; 95% CI 1.7–21.0), and symptom duration shorter than 24 h (aOR 3.8; 95% CI 1.1–13.1) as independent factors associated with death (15). More recently, a Greek pediatric cohort reported that mortality was significantly associated with meningococcemia presentation, abnormal platelet count at admission, ICU admission, and coagulation disorders (23). Although these studies used different outcomes and populations, they consistently show that early systemic compromise and rapid disease progression are important determinants of poor outcome. In our cohort study, the most consistent findings seizure during hospitalization, prolonged capillary refill, and petechial or purpuric rash were associated with higher odds of septic shock in the parsimonious Firth model. These associations were also observed in the sensitivity analysis (Supplementary Table S1).

Irritability or drowsiness was markedly more frequent among patients with an adverse outcome in the unadjusted analysis, and a lower median GCS score was also observed in this group. However, the adjusted estimate for this variable was imprecise in the primary admission model. This attenuation may reflect overlap with seizure and other neurological severity indicators, together with the limited amount of information available for estimating several correlated effects. The finding should therefore not be interpreted as evidence that altered mental status lacks clinical importance; rather, it illustrates the distinction between a strong univariable association and an independently estimated coefficient in a small multivariable model.

Clinical presentation also played a critical role in the observed outcome. A consistent finding in our study was the difference in clinical outcome according to disease presentation, with isolated meningitis showing a more favorable outcome profile than meningococcemia in univariable association (*p* = 0.001). Indeed, all recorded fatalities and most episodes of septic shock occurred among children with meningococcemia, either alone or combined with meningitis, whereas no deaths were observed in patients with isolated meningitis. This is consistent with the results of a large retrospective study from Romania including 256 patients across all age groups that reported a CFR of 30% in meningococcemia, compared with 2% in isolated meningitis and 6% in meningitis with meningococcemia, with statistically significant differences (*p* < 0.001) (27). A similar gradient was reported in a Brazilian cohort from Recife, with case fatality rising from 8.7% in isolated meningitis to 35.2% in meningitis with meningococcemia and 62.1% in meningococcemia alone (15). Poulikakos et al. (23) reported similar observations in a multicenter study in Greece in children with IMD, who demonstrated that meningococcemia was independently associated with both higher mortality and increased risk of severe complications, while isolated meningitis was associated with more favorable survival and neurological outcomes. Despite a high burden of neurological symptoms in meningitis cases, the absence of profound hemodynamic compromise may translated into better short-term survival (23, 28). Nevertheless, in our study the apparent inverse association between isolated meningitis and the outcome remained uncertain after adjustment in the extended model, so rather than suggesting a direct biological protective effect of meningeal involvement, this finding more likely reflects meaningful differences in clinical presentation, extent of systemic involvement and timing of diagnosis.

In Chile, a previous epidemiological study identified factors associated with acquiring IMD identified age <1 year, household crowding, close interpersonal contact, and prior hospitalization for pertussis as significant risk factors for developing IMD in children under 5 years (16). In contrast, our study evaluated clinical factors associated with severe outcome after IMD had already been established, thereby complementing previous epidemiological evidence with information relevant to early clinical risk assessment.

In the univariable analysis, serogroup distribution differed between outcome groups: MenB was more frequent among children who developed septic shock (57.1% vs. 22.7%), whereas MenW predominated among those without adverse outcome (61.4% vs. 32.1%). However, MenB was not independently associated with the outcome in either the extended model or the multiple imputation analysis, and information on serogroup was unavailable for 8 of 72 patients. Therefore, the observed differences may reflect confounding by patient characteristics, disease severity, or circulating meningococcal lineages rather than an independent effect of capsular serogroup. In a systematic review with meta-analysis (24), MenB was associated with the lowest pooled CFR of 6.9% compared to non-B serogroups, which exhibited higher raw mortality: MenW 12.8%, MenC 12.0%, and MenY 10.8%. Furthermore, multiple prospective cohorts, including the Canadian IMPACT registry (29) and the Dutch national surveillance (30), both with more than 800 cases, reported that capsular serogroups do not exert any independent effect on mortality or long-term complications after adjustment for clonal complex (cc) and clinical severity at admission.

From a public health perspective, our results emphasize the importance of early clinical recognition of IMD, particularly in children who present without classical meningeal signs. Non-meningeal presentations can be especially challenging, as they often begin with nonspecific symptoms (16, 31). In our patients, gastrointestinal symptoms were common and occurred across all clinical phenotypes, reinforcing their limited discriminatory value and potential role in delaying diagnosis (16, 31, 32). In contrast, seizure during hospitalization, prolonged capillary refill, and petechial or purpuric rash were associated with septic shock and may help identify children with a high-risk clinical presentation.

The present study had several limitations. First, as a retrospective observational study based on medical records, the associations identified cannot be interpreted as causal relationships and residual confounding cannot be excluded. Second, the cohort was restricted to 72 patients younger than 15 years from two tertiary pediatric hospitals in Santiago (HLCM and HEGC), representing 22.6% of pediatric IMD notifications in the Santiago Metropolitan Region during 2009–2025 and corresponding to approximately 60% of notifications within the nominal referral areas of these hospitals (East and South, respectively). However, because referrals across health-service areas may occur and individual referral pathways could not be reconstructed, selection bias and limited generalizability remain possible. Third, a key microbiological limitation is the lack of access to molecular characterization data of most meningococcal isolates, such as cc, serotypes, or serosubtypes, because of that we were unable to determine their relationship with patient outcome. Fourth, due to only five deaths occurring, the study lacked sufficient events to build a reliable mortality-specific multivariable model. However, all fatal cases occurred among patients who developed septic shock. Therefore, mortality was assessed descriptively, while septic shock was examined using multivariable modeling. Fifth, the number of adverse outcomes was small relative to the number of potential covariates. Firth regression reduces sparse-data bias and resolves infinite estimates under separation, but it does not eliminate overfitting, post-selection uncertainty, or instability from stepwise procedures (33–35). The broad CIs, attenuation of some coefficients across models, and variable bootstrap selection frequencies are direct evidence of residual uncertainty. The analysis was consequently kept parsimonious and should be considered exploratory rather than a validated prediction model. Sixth, lactate was missing in 24/72 patients. Multiple imputation allowed its inclusion in a sensitivity analysis, but the validity of that analysis depends on the imputation model and the assumption that missingness was explainable by observed data (35). Finally, the long study period also included changes in vaccination policy, diagnostic practice, and meningococcal epidemiology that could not be modelled reliably with this sample size. The observed decline after 2014 is temporally consistent with the national reduction in MenW incidence reported following MCV-ACWY introduction in Chile’s toddler immunization program (36–38). However, this two-center retrospective cohort was not designed to estimate vaccine effectiveness or population-level impact.

Despite these limitations, the use of microbiologically confirmed cases, the inclusion of two referral hospitals, the explicit separation of admission variables from later complications, and the sensitivity analyses strengthen the interpretation. The findings identify clinically plausible factors associated with severe outcome in this cohort and support confirmation in larger, multicenter prospective studies with standardized timing of measurements and molecular characterization.

In conclusion, although IMD is preventable through vaccination, it continues to represent a considerable clinical and public health burden. Our results emphasize the relevance of maintaining a high level of clinical suspicion, particularly in those who do not present classic meningeal features. Maintaining robust surveillance systems, ensuring high vaccine coverage, and promoting early recognition at the community and hospital levels remain critical strategies to reduce mortality and improve outcomes.

## Data Availability

The datasets presented in this article are not readily available because anonymized datasets generated and/or analyzed during the current study may be made available from the corresponding author upon reasonable request, subject to ethical approval and institutional regulations. No participant-identifiable data will be shared. Requests to access the datasets should be directed to msantola@uchile.cl.
