# Supplementary material for: Clinical factors associated with septic shock in pediatric invasive meningococcal disease: a retrospective study from two tertiary hospitals in Chile, 2009–2025
Source: Front Public Health. 2026 Oct 13;14:1911106. doi: 10.3389/fpubh.2026.1911106 (PMC13710219; doi:10.3389/fpubh.2026.1911106)
Supplement: Supplementary file 1 [file Data_Sheet_1.PDF]

CLINICAL FACTORS ASSOCIATED WITH SEPTIC SHOCK IN PEDIATRIC INVASIVE MENINGOCOCCAL DISEASE: A RETROSPECTIVE STUDY FROM TWO TERTIARY HOSPITALS IN CHILE, 2009-2025.

Supplementary material

List Tables

Table S1. Sensitivity analysis evaluating robustness and stability of the multivariable findings (N=72)

List Figures

Figure S1. Phi correlations among selected candidate variables considered during model development
Figure S2. Data availability for candidate variables considered during model development

Table S1. Sensitivity analysis evaluating robustness and stability of the multivariable findings (N=72)

**Variable/Analysis**	**Extended multivariable model***	**Alternative Firth model**, aOR (95% CI)**	**Leave-one-out analysis***, OR range**	**Multiple-imputation analysis****, aOR (95% CI)**
Seizure during hospitalization	15.34 (2.86-122.42)	-	9.41-18.07	9.07 (1.45-56.81)
Prolonged capillary refill	5.71 (1.31-31.16)	6.64 (1.62-33.07)	4.62-8.94	7.70 (1.42-41.92)
Irritability / drowsiness	1.95 (0.37-10.81)	2.22 (0.45-11.56)	2.05-3.67	1.45 (0.23-9.22)
Petechial or purpuric rash	2.88 (0.76-12.16	6.71 (1.70-33.64)	3.88-6.94	-
Lactate > 20 mg/dL	-	-	-	4.88 (0.97-24.60)
Serogroup B	2.77 (0.72-11.55)	-	-	4.81 (0.97-24.60)
Isolated meningitis	0.21 (0.03-1.05)	-	-	0.23 (0.04-1.36)
Seizure at admission	-	23.98 (4.07-203.77)	-	-

* Extended multivariable model included variables from final model with Serogroup B with isolated meningitis.
** Alternative Firth model using seizures at admission instead of seizures occurring during hospitalization.
*** Leave-one-out analysis in which the primary Firth model was refitted after sequentially excluding one patient at a time; values represent the minimum and maximum ORs across iterations.
**** Multiple-imputation analysis using chained equations with 50 imputed datasets; pooled aORs and 95% CIs are reported.
aOR, adjusted odds ratio; OR, odds ratio; CI, confidence interval.

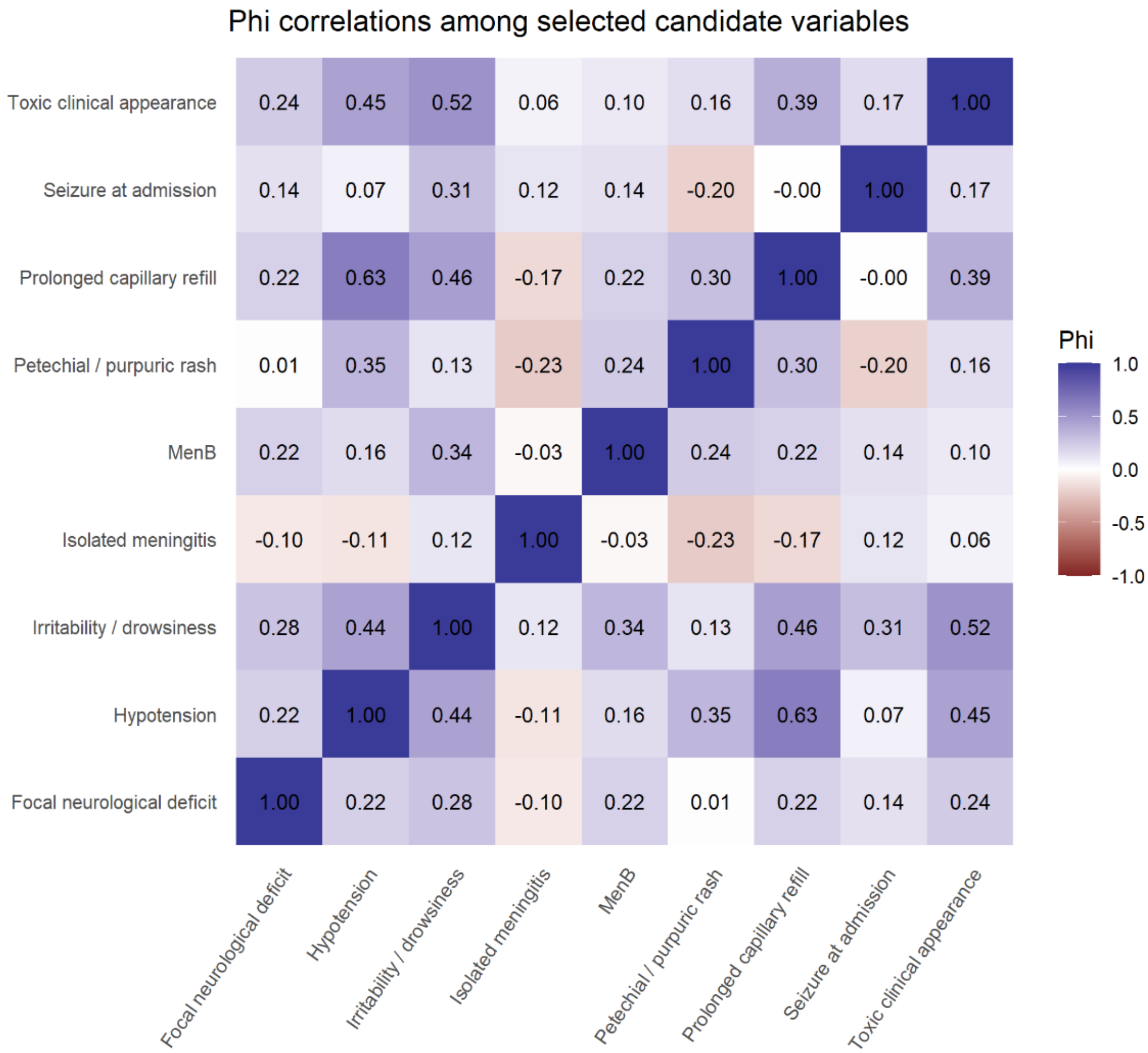

Figure S1. Phi correlations among selected candidate variables considered during model development. Phi coefficients were calculated for selected binary variables to assess collinearity and conceptual overlap. The highest correlation was observed between hypotension and prolonged capillary refill (phi = 0.63); no pair showed a correlation suggestive of severe collinearity.

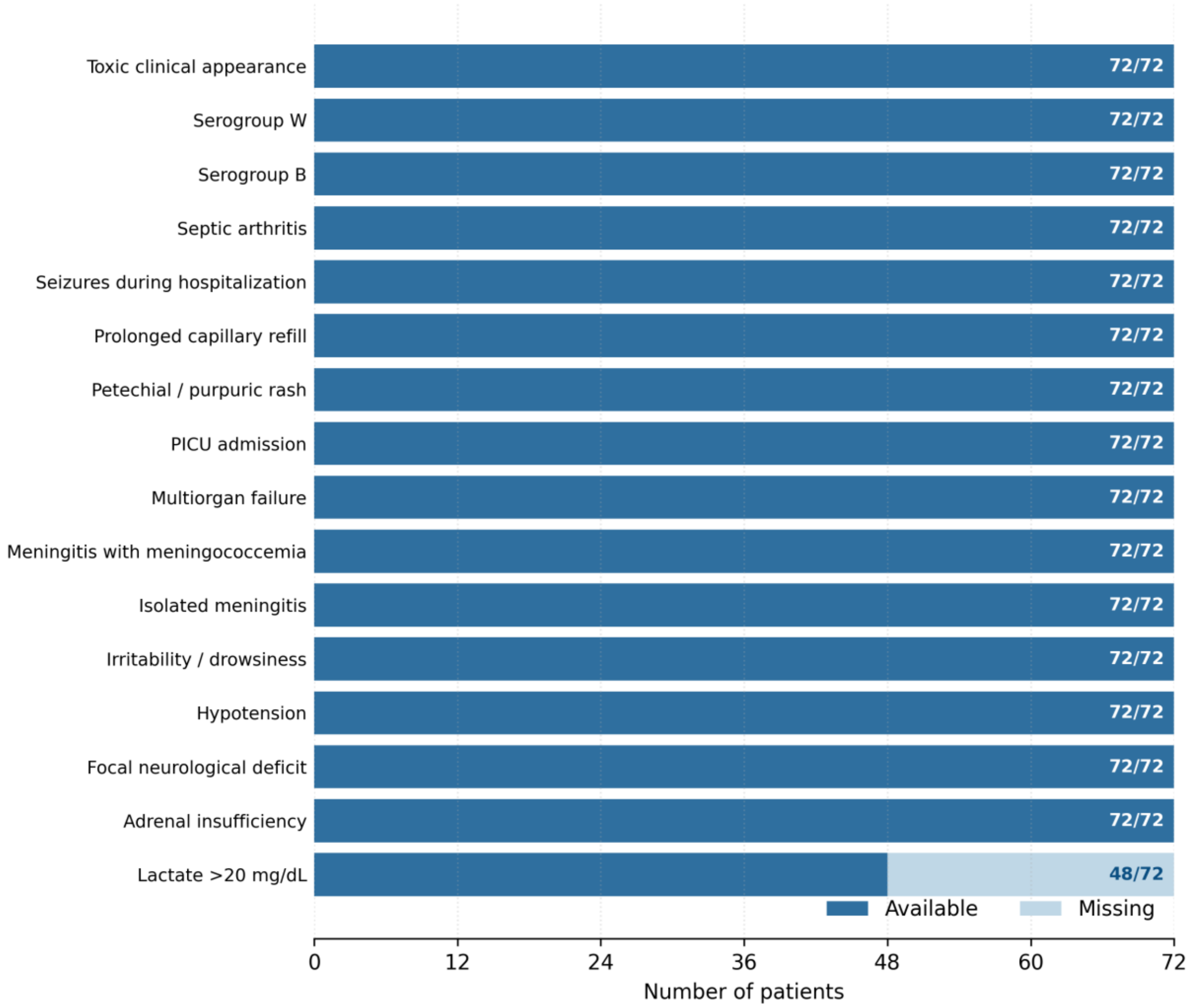

Figure S2. Data availability for candidate variables considered during model development. Available and missing observations are shown for candidate variables considered during model development. All variables had complete data except lactate >20 mg/dL, which was available for 48 of 72 patients.
